# Maternal Alexithymia and Attachment Style: Which Relationship with Their Children’s Headache Features and Psychological Profile?

**DOI:** 10.3389/fneur.2017.00751

**Published:** 2018-01-22

**Authors:** Samuela Tarantino, Laura Papetti, Cristiana De Ranieri, Francesca Boldrini, Angela Maria Rocco, Monica D’Ambrosio, Valeria Valeriano, Barbara Battan, Maria Francesca Paniccia, Federico Vigevano, Simonetta Gentile, Massimiliano Valeriani

**Affiliations:** ^1^Division of Neurology, Headache Center, Bambino Gesù Ospedale Pediatrico (IRCCS), Rome, Italy; ^2^Unit of Clinical Psychology, Ospedale Pediatrico Bambino Gesù (IRCCS), Rome, Italy; ^3^Center for Sensory-Motor Interaction, Aalborg University, Aalborg, Denmark

**Keywords:** children, attachment style, migraine severity, psychological factors, mothers, alexithymia

## Abstract

**Introduction:**

A growing body of literature has shown an association between somatic symptoms and insecure “attachment style.” In a recent study, we found a relationship between migraine severity, ambivalent attachment style, and psychological symptoms in children/adolescents. There is evidence that caregivers’ attachment styles and their way of management/expression of emotions can influence children’s psychological profile and pain expression. To date, data dealing with headache are scarce. Our aim was to study the role of maternal alexithymia and attachment style on their children’s migraine severity, attachment style, and psychological profile.

**Materials and methods:**

We enrolled 84 consecutive patients suffering from migraine without aura (female: 45, male: 39; mean age 11.8 ± 2.4 years). According to headache frequency, children/adolescents were divided into two groups: (1) high frequency (patients reporting from weekly to daily attacks), and (2) low frequency (patients having ≤3 episodes per month). We divided headache attacks intensity into two groups (mild and severe pain). SAFA “Anxiety,” “Depression,” and “Somatization” scales were used to explore children’s psychological profile. To evaluate attachment style, the semi-projective test SAT for patients and ASQ Questionnaire for mothers were employed. Maternal alexithymia traits were assessed by TAS-20.

**Results:**

We found a significant higher score in maternal alexithymia levels in children classified as “ambivalent,” compared to those classified as “avoiding” (Total scale: *p* = 0.011). A positive correlation has been identified between mother’s TAS-20 Total score and the children’s SAFA-A Total score (*p* = 0.026). In particular, positive correlations were found between maternal alexithymia and children’s “Separation anxiety” (*p* = 0.009) and “School anxiety” (*p* = 0.015) subscales. Maternal “Externally-oriented thinking” subscale correlated with children’s school anxiety (*p* = 0.050). Moreover, we found a correlation between TAS-20 Total score and SAFA-D “Feeling of guilt” subscale (*p* = 0.014). Our data showed no relationship between TAS-20 and ASQ questionnaires and children’s migraine intensity and frequency.

**Conclusion:**

Maternal alexithymia and attachment style have no impact on children’s migraine severity. However, our results suggest that, although maternal alexithymic traits have no causative roles on children’s migraine severity, they show a relationship with patients’ attachment style and psychological symptoms, which in turn may impact on migraine severity.

## Introduction

Migraine is a complex disease and the underlying pathogenetic mechanisms are not completely understood. Since the past decades, many authors have evidenced a relationship between migraine and psychological factors both in adult (1, 2) and in pediatric age (2, 3); nevertheless, the exact nature of this relationship remains unclear (2, 3). So far, much remains to be learned about contextual psychological, environmental, and interpersonal vulnerability factors that may contribute to this link.

There is evidence that parent–child interaction may impact child’s personality, psychological, and physical development (4). As a result of early experiences and interactions with primary caregivers, children develop cognitive schemas regarding the self and others (internal working models) that influence thoughts, emotional responses, and interpersonal relationships throughout their life (5, 6). Four patterns of attachment have been identified in children (Table 1). Based on all the possible combinations of the internal models of others (positive/negative) and self (positive/negative), Bartholomew and Horowitz (7) were the first to describe a four factors classification system of adult attachment styles (Table 2).

**Table 1:** Attachment styles in children/adolescents.

Attachment style	Caregiver behavior	Children view of self/behavior
Secure	Consistently responsive and in tune with the child’s emotions. Attachment figure is seen as a source of comfort and reassurance	Children believe and trust that his/her need will be met. Cognitive representations of self and of others are positive. Positive social self-efficacy

Insecure–ambivalent	Predictably unpredictable, which is sometimes responsive and sometimes not responsive to negative emotional signals	Poor self-esteem, increased subjective distress, and increased focus on negative affect. Children show exaggerated non-verbal affective signals to “coerce” their unpredictable parents to respond in a particular way

Insecure–avoiding	Consistently unresponsive. The caregiver predictably responds with withdrawal or anger when the child is distressed	Subconscious believes that his/her needs probably will not be met. Children implicitly learn to inhibit signals of distress or anger because they are not useful in obtaining comfort. Nevertheless, they have a positive view of themselves, resulting in self-reliance

Insecure–disorganized/confused	Extremely unattached or malfunctioning	Poor self-esteem, more subjective distress, and increased vigilance of negative affect. Severely confused with not strategy to have theirs needs met. Children share many of the characteristics of preoccupied individuals in that they desire social contact, but this desire is ultimately inhibited by fear of rejection
	The attachment figure is seen as frightening	

**Table 2:** Adult attachment style according to Bartholomew and Horowitz’s model.

	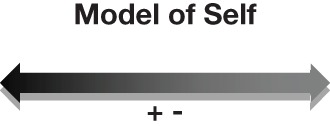	
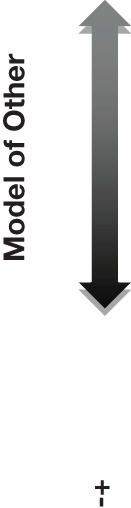	*Secure*: high self-worth, believes that others are responsive, comfortable with autonomy and in forming close relationships with others	*Preoccupied*: a sense of self-worth that is dependent on gaining the approval and acceptance of others

	*Dismissing*: overt positive self-view, denies feelings of subjective distress and dismisses the importance of close relationships	*Fearful*: negative self-view, lack of trust in others, subsequent apprehension about close relationships and high levels of distress

Evidence from recent studies suggests that children’s attachment style might play a relevant role in pain experience (8–10). Several studies showed that the model of self and others, the self-sabotaging attitude, the negative cognitive orientations, and emotional distress, typical of insecure attachment, may impact on frequency, severity, and management of pain, both in adult (11, 12) and pediatric age (5, 8, 13).

In a recent study, we evidenced a high prevalence of ambivalent attachment style among young migraineurs; in particular, our data showed an association between migraine features (frequency and intensity of attacks), ambivalent attachment style, and psychological symptoms (14). These findings supported the hypothesis that a dysfunctional parent–child interaction may be a common vulnerability factor for both psychological symptoms and headache severity in children/adolescents suffering from migraine (14).

Parental responses to children’s emotional expressions have been often taken into account, trying to predict their effects on child’s developmental outcomes; according to recent empirical pieces of evidence, parents’ response shows a great variability with relevant implications on offspring’s socio-emotional competences, pro-social behavior, attachment style, regulation of affective responses, and coping (15). Parental responsiveness to their children’s emotions may vary substantially ranging from being sensitive and supportive to minimizing or amplifying children’s distress and pain signals. Parental responsiveness, in turn, may impact on children emotional expression and somatic illness (8). Underline factors that explain parental sensitivity and responses to children’s emotional signals may be predicted by their own attachment style (15, 16). There is evidence that maternal attachment style may influence their response to their children’s (17, 18) or adolescents’ (15) negative emotions. In particular, data from the literature showed the role of maternal emotion regulation as important mediating mechanism between attachment and caregiving responses (15). Parents with an anxious attachment orientation may use hyperactivating strategies of dealing with distress (19), adopting strategies focused on negative emotions for both their own and their children’s distress; on the other hand, parents with an avoidant attachment may imply deactivating strategies and emotional inhibition to cope with stressful situations and negative emotions (19). Thus, as a result of early interaction with their caregiver, children may implicitly learn to amplify or suppress their emotions and body signals (8). While several studies analyzed the impact of maternal attachment insecurity on their children’s psychological symptoms (sleep disorders, behavior problems) (20, 21), few studies focused on the effect on children/adolescents’ somatic symptoms (22). To the best of our knowledge, no study explored maternal attachment style in children/adolescents suffering from migraine.

One additional maternal feature which may also be correlated with low maternal care is her dysfunction in emotional awareness (alexithymia) (23, 24). Alexithymia is a personality trait characterized by an impaired ability to identify and communicate emotions, difficulty in differentiating between feelings and body sensations, an externally oriented cognitive style and limited imagination (25). Two further elements of alexithymia are detectable: an affective one, according to which people might have difficulties in responding to, feeling and sharing emotions, as well as a cognitive component in relation to which people might have difficulty in understanding their own feelings and trying to talk about emotions (26). Several studies have described the relationship between alexithymic traits, psychiatric, and somatic symptoms (27, 28); by contrast, the potential influence of parental, in particular maternal, alexithymia on children’s developmental pathways, psychological symptoms, and health is still not clarified. In a recent paper, Paniccia et al. (29) found significant levels of alexithymia in mothers of adolescents with generalized anxiety disorder. Although some authors explored the role of maternal alexithymic traits on children’s health, studies investigating the correlations between mothers’ alexithymia and children’s somatic symptoms and pain are scarce. There are pieces of evidence suggesting that alexithymic traits are common in mothers (and fathers) of female patients affected by eating disorders (30, 31). Previous data showed a relationship between maternal alexithymia and children/adolescents’ diabetic control (32). So far, data exploring maternal alexithymic traits in children with migraine are limited (33, 34). In a recent study, Cerutti et al. explored the relationship between migraine and alexithymia levels in adolescents and their mothers, as well as the influence of this link on possible psychopathological symptoms (both in patients and their mothers) (33). The abovementioned study, however, did not analyze the effect of maternal alexithymia on their children’s migraine severity.

Here, we evaluated a selected population of children/adolescents suffering from migraine without aura together with their mothers. Aims of our study were to explore the role of maternal attachment style and alexithymia on (1) their children’s headache severity (intensity and frequency) and (2) their children’s attachment style and psychological profile (anxiety, depression, somatization) (35).

## Materials and Methods

### Subjects and Procedures

The present study was carried out from May 2012 to October 2014. We enrolled 84 consecutive patients suffering from migraine without aura (female: 45, male: 39; age range 8–18 years; mean age 11.8 ± 2.4 years) (35). Children/adolescents were systematically referred for consultation at our Headache Center.

The International Classification of Headache Disorders Criteria, 3rd edition (ICHD-III-beta) were used for migraine diagnosis (36). Patients suffering from any other organic or neurological disease were excluded from the study.

All our patients had to complete a headache diary, in which they had to sign the main features of headache (such as intensity and frequency of the attacks): the diary was given at the initial visit and was brought back by families at the second consultation (2 months after the initial visit).

According to the headache frequency, patients were divided into two groups: (1) high frequency (HF; patients reporting from weekly to daily attacks) and (2) low frequency (LF) (patients having ≤3 episodes per month) (35).

Several reasons justified the setting of the above mentioned break point: (1) the number of patients with chronic and intermediate frequencies was too limited to allow reliable statistic analysis; (2) the distinction between chronic and episodic patients would have led to the inclusion of subjects with HF of attacks, but not chronic, in the same group of very low attack frequency patients; (3) patients who need prophylactic treatment were distinguished from those who do not (37). We classified patients in two attack intensity groups: (1) severe pain (SP), leading to interruption of patient activities or forcing the child to go to bed and (2) mild pain (MP), allowing child/adolescent to continue his/her daily activities (35).

None of the patients had been treated with medications acting on the central nervous system (including psychiatric treatments) and none of them was receiving prophylactic medications for migraine. Assumption of symptomatic drugs (i.e., ibuprofen, paracetamol) for acute attacks did not contraindicate the inclusion in the study.

The same examiner (Samuela Tarantino—with a specific training on the psychological assessment of children/adolescents and the attachment theory) performed the psychological evaluation in a single session; the screening was made of a psychological interview and the administration of various psychological tests.

At the moment of the psychological screening, the examiner was blind of headache features (frequency and intensity of the migraine attacks).

In order to exclude possible effects of pain on the psychological assessment, patients who suffered headache attack within 24 h before the psychological study were excluded from our sample.

An informed consent was signed by all participants and by their parents. The study was approved by the local Ethics Committee of the Ospedale Pediatrico Bambino Gesù, in compliance with the Helsinki Declaration.

### Measures

– The attachment style was analyzed by the SAT (Separation Anxiety Test, modified Italian version) (38–40). The SAT is a semi-structured interview that explores children’s feelings and thoughts regarding attachment patterns. In this version, children are invited to see six pictures (gender-matched) that represent separations between a child and his/her parent(s).

In order to avoid any undue pressure on the children with anxiety and negative-inducing emotions, two types of scenes (severe and mild parent–child separation) are showed alternately. The pictures display the following scenes: (1) mother and father go out for the evening and leave the child at home (mild scene); (2) on the first day of school, the mother drops off the child (mild scene); (3) mother and father go away for a weekend and leave their child with his/her aunt (severe scene); (4) parents are talking and ask the child to walk away from them (mild scene); (5) parents give a present to the child before leaving him/her for 2 weeks (severe scene); and (6) mother leaves the room after having tucked the child in bed (mild scene). The examiner explains each picture and asks to the child three questions: (1) how does the boy/girl in the picture feel? (2) why do you think the child feels happy, sad, mad, etc? and (3) what the boy/girl will do or will say?. The total score is composed by the sum of two global ratings: emotional security and quality of coping responses. Children who are unwilling to show vulnerability, deny the separation or show bizarre or disorganized behavior, obtain low emotional security scores; high security scores are given to children who show recognition of attachment feelings and the corresponding motives. Low coping scores reflect maladaptive coping strategies (e.g., start eating, run away, and hurt someone) or the absence of coping strategy (e.g., do nothing or wait for his/her parents); on the other hand, high coping scores reflect adaptive or constructive strategies involving positive separation behaviors or social support (e.g., stay with the baby sitter or watch TV).

According to the total score, attachment style is classified in (1) secure attachment style (score +4 and above); (2) ambivalent attachment style (score +1 to +3); (3) avoidant attachment style (score −2 to 0); and (4) disorganized/confused attachment style (score −3 and below). SAT shows a satisfactory test–retest reliability and demonstrates a good inter-rater reliability (Cohen’s kappa: 0.80) (38).

– To assess the psychological symptoms, the Italian SAFA battery of tests (Self-Administrated Psychiatric Scales for Youths and Adolescents) was employed (41). It allows to explore a wide series of psychiatric symptoms and disorders. The battery is composed by a total of six scales (each with subscales): SAFA-A (anxiety-related symptoms), SAFA-S (somatic concerns), SAFA-D (depression-related symptoms), SAFA-O (obsessive–compulsive symptoms), SAFA-P (psychogenic eating disorders), and SAFA-F (phobias). SAFA provides a general profile and/or individual profiles within the single scales (which can also be used separately). The administration lasts between 30 and 60 min. The SAFA battery embraces subjects from 8 to 18 years old: each questionnaire includes a version for children ranging from 8 to 10 years (identified with the letter “e”) and a version for subjects aged from 11 to 18 years (“ms”). Only SAFA-A has three distinct versions: 8–10 years (“e”), 11–13 years (“m”), and 14–18 years (“s”). Children/adolescents evaluate each item according to three possible responses: “true (scored 2), partly true (scored 1), and false (scored 0)”; scores obtained in each scale and subscale can be converted into *T* scores, percentiles, and sten points. The scales showed good test–retest stability and internal consistency (Cronbach alpha >0.80). The psychometric properties have been described for each scale (41).
  In the present study, we administered SAFA-A (anxiety), SAFA-S (somatic concerns), and SAFA-D (depression) scales. SAFA-A evaluates generalized anxiety, social anxiety, separation anxiety, and school anxiety symptoms. SAFA-D includes “Depressed mood,” “Anhedony/disinterest,” “Touchy mood,” “Sense of inadequacy/low self-esteem,” “Insecurity,” “Feeling of guilt,” and “Hopelessness” subscales. No items explored symptoms, such as weight variation, sleep problems, asthenia, and concentration difficulties, which are included in SAFA-S scale. In particular, SAFA-S explores somatic symptoms and hypochondria.
– To assess maternal alexithymia, the TAS-20 (Toronto Alexithymia Scale) was used. The TAS-20 is a self-report measure of alexithymia, composed by 20 items. It assesses both the affective and cognitive elements of the alexithymia construct (42, 43). Subjects judge each item according to the five-point likert scale. The total score ranges from 20 to 100 (higher scores indicate greater levels of alexithymia). In addition to a total score, the TAS-20 provides three factor scores: (1) TAS-F1, “Difficulty identifying feelings” (contains seven items and analyzes difficulties in identifying and differentiating physical sensations from emotions); (2) TAS-F2, “Difficulty describing feelings” (it contains five items and explores difficulties in verbally communicating feelings), and (3) TAS-F3, “Externally oriented thinking” (contains eight items and measures the tendency to be focused on details of external events rather than on internal and intimate emotional experiences). Evidence of acceptable internal consistency, test–retest reliability, and construct, concurrent, and convergent validity has been reported (42, 43).
– To evaluate maternal attachment, we employed the ASQ (Attachment Style Questionnaire) (44, 45). The ASQ is a self-report questionnaire developed to explore adult attachment dimensionally rather than categorically. It consists of 40 items, with response options ranging from 1 (totally disagree) to 6 (totally agree). The test explores general relationships rather than close or romantic relationships. The ASQ includes five scales: (1) ASQ-F1, “Confidence in relationships”; higher scores in this subscale indicate a secure attachment (e.g., “I find it relatively easy to get close to other people”); (2) ASQ-F2, “Need for approval” denotes both worried and fearful aspects of attachment, characterized by an individual’s need for others’ approval and acceptance (e.g., “It’s important for me to avoid doing things that others won’t like”); (3) ASQ-F3: the subjects’ anxious behavior in searching for others, motivated by the necessity to fulfill dependency needs, is depicted by the subscale “Preoccupation with relationships”; it represents a central topic in the conceptualization of anxious/ambivalent attachment (e.g., “It’s very important for me to have a close relationship”); (4) ASQ-F4, “Discomfort with closeness” reflects an avoidant attachment (e.g., “ I prefer to keep to myself”), and (5) ASQ-F5 “Relationships as secondary” is typical of a dismissive style, in which subjects tend to emphasize achievements and independence, in order to protect themselves against hurt and vulnerability (e.g., “To ask for help is to admit that you’re a failure”). The questionnaire has acceptable levels of test–retest reliability and high levels of internal consistency. Reliability and validity data have been provided for both English (44) and Italian (45) versions of the ASQ.

### Statistical Analysis

Statistical analysis was performed using SPSS 22.0 software (Statistical Package for the Social Sciences). According to the aim of our study, patients were grouped according to pain severity (MP and SP groups), attack frequency (LF and HF), and attachment styles (secure, ambivalent, avoidant, and disorganized/confused). Initially, we analyzed the frequencies of each category of variables (frequency of attacks, intensity of pain, and attachment styles).

ANOVA was performed to assess if maternal alexithymia and attachment style were different in relation to their children’s headache frequency and intensity. We compared every TAS-20 (TAS-F1-F3) and ASQ (ASQ-F1-F5) factors among the different groups of patients (MP/SP, LF/HF). Moreover, to explore differences in maternal alexithymia and attachment as function of children/adolescents’ attachment style (secure, ambivalent, avoiding, and disorganized/confused) a series of one-way ANOVAs was carried out. We used Bonferroni’s test for the *post hoc* analysis.

A series of bivariate correlation with Pearson test was carried out to analyze correlations between TAS-20 and ASQ factors and their children’s SAFA-A, D, and S scores.

Statistical significance was determined by two-tailed test and *p*-value was fixed at *p* < 0.05.

## Results

### Headache Features

Most patients had a HF of attacks (54.8%), while 38 patients (45.2%) suffered from LF episodes. Most children described pain as severe (63.1%); headache pain was mild/moderate in 36.9% of the children. Headache clinical features of our patients are summarized in Table 3.

**Table 3:** Headache characteristics of our sample.

	*N* = 84
**Pain intensity**	
Mild	31 (36.9%)
Severe	53 (63.1%)
**Frequency**	
Low frequency	38 (45.2%)
High frequency	46 (54.8%)
**Associated symptoms**	
Nausea	39 (46.4%)
Vomiting	22 (26.2%)
Phonophobia	62 (73.8%)
Photophobia	54 (64.3%)

### Patients’ Attachment Styles Distribution

We found a high prevalence of the insecure attachment style (88.1%). Ambivalent attachment style was found in 42.9% of the patients, while 36.9% of the children were classified in the avoidant group. A smaller number of patients were classified as disorganized/confused attachment style (8.3%) (Table 4).

**Table 4:** Attachment styles distribution among our migraine children/adolescents.

Attachment styles	*n* = 84	%
Secure	10	11.9
Insecure	74	88.1
Ambivalent	36	42.9
Avoiding	31	36.9
Disorganized/confused	7	8.3

### Role of Maternal Alexithymia and Attachment Style on Their Children Migraine Features

TAS-20 Total scores were not significantly related to intensity (MP/SP, *F* = 2.546; *p* = 0.114) and frequency (LF/HF, *F* = 0.838; *p* = 0.363) of patients’ headache. When we analyzed TAS-20 “Difficulty identifying feelings” (TAS-F1), “Difficulty describing feelings” (TAS-F2), and “Externally oriented thinking” (TAS-F3) subscales, we found no significant effect on intensity and frequency of our patients’ migraine. No statistically significant relationships were found between maternal ASQ subscales scores and their children’s severity of migraine (frequency and intensity) (Table 5).

**Table 5:** Maternal TAS-20, ASQ scores (mean ± SD), and ANOVA among children frequency/intensity based groups.

	HF	LF	*F* value	*p*
TAS-Tot	44.28 ± 12.814	41.79 ± 11.939	0.838	0.363
TAS-F1	22.87 ± 6.581	20.66 ± 8.802	1.733	0.192
TAS-F2	22.07 ± 7.052	19.32 ± 8.804	2.525	0.116
TAS-F3	17.04 ± 5.134	17.21 ± 4.173	0.026	0.872
ASQ-F1	35.11 ± 5.539	35.89 ± 5.208	0.442	0.508
ASQ-F2	19.37 ± 6.020	20.26 ± 7.020	0.394	0.532
ASQ-F3	25.04 ± 7.554	24.08 ± 7.872	0.327	0.569
ASQ-F4	31.61 ± 8.531	30.84 ± 9.140	0.158	0.692
ASQ-F5	15.89 ± 5.740	15.68 ± 5.393	0.029	0.866

	**MP**	**SP**	***F* value**	***p***

TAS-Tot	44.79 ± 11.593	40.35 ± 13.440	2.543	0.114
TAS-F1	21.13 ± 8.043	23.13 ± 7.013	1.322	0.254
TAS-F2	19.96 ± 8.295	22.29 ± 7.263	1.685	0.198
TAS-F3	17.83 ± 4.397	15.90 ± 5.009	3.387	0.069
ASQ-F1	35.28 ± 5.361	35.77 ± 5.469	0.162	0.689
ASQ-F2	19.70 ± 6.721	19.90 ± 6.112	0.019	0.889
ASQ-F3	25.06 ± 8.108	23.84 ± 6.909	0.490	0.486
ASQ-F4	32.19 ± 9.556	29.68 ± 7.087	1.617	0.207
ASQ-F5	16.17 ± 5.676	15.16 ± 5.367	0.642	0.425

*p ≤ 0.05.

HF, high frequency; LF, low frequency; MP, mild pain intensity; SP, severe pain intensity; TAS-F1, “Difficulty identifying feelings,” TAS-F2, “Difficulty describing feelings,” and TAS-F3 “Externally oriented thinking”; ASQ-F1, “Confidence in relationships”; ASQ-F2, “Need for approval”; ASQ-F3, “Preoccupation with relationships”; ASQ-F4, “Discomfort with closeness”; ASQ-F5, “Relationships as secondary.”

Our data showed no relationship between maternal alexithymia, attachment style, and children’s migraine severity.

### Relationship between Maternal Alexithymia, Attachment Style, and Children’s Attachment Style

When we compared TAS-20 Total scores according to patients’ attachment style, a significant difference among groups was found (TAS-20, *F* = 3.838; *p* = 0.013). *Post hoc* analysis showed a significantly higher score in mothers whose children were classified as “ambivalent” attached, compared to those classified as “avoiding” (TAS-20 Total: *p* = 0.011) (35). TAS-20 factors “Difficulty identifying feelings” (TAS-F1) (*F* = 2.264; *p* = 0.087), “Difficulty describing feelings” (TAS-F2) (*F* = 2.098; *p* = 0.107), and “Externally-oriented thinking” (TAS-F3) (*F* = 1.200; *p* = 0.315) did not show any significant difference among children/adolescents attachment styles. The ASQ subscales showed no significant relationship with patients’ attachment styles.

### Influence of Maternal Attachment and Alexithymia Levels on Their Children/Adolescents’ Psychological Profile

TAS-20 Total score showed a significant and positive correlation with SAFA-A “Separation anxiety” (*r* = 0.283; *p* = 0.009), “School anxiety” (*r* = 0.264; *p* = 0.015) subscales and with “Total anxiety” (*r* = 0.244; *p* = 0.026) scales. Moreover, we found a correlation between TAS-20 Total score and SAFA-D “Feeling of guilt” subscale (*r* = 0.283; *p* = 0.014) (Table 6). Analyzing all TAS-20 factors, we found a significant relationship between TAS-20 “Externally-oriented thinking” (TAS-F3), children/adolescents’ school anxiety (SAFA-A) (*r* = 0.214; *p* = 0.050) and feeling of guilt (SAFA-D) (*r* = 0.235; *p* = 0.044). Furthermore, ASQ analysis showed a negative relationship between “Confidence” (in self and in others) factor and “School anxiety” (*r* = −0.214; *p* = 0.050) (Table 7) (35).

**Table 6:** Correlation between TAS-20 and SAFA-A, S, and D.

	TAS-Tot	TAS-F1	TAS-F2	TAS-F3
SAFA-A Tot	*r* = 0.244	*r* = 0.091	*r* = 0.057	*r* = 0.095
	*p* = 0.026*	*p* = 0.412	*p* = 0.607	*p* = 0.388
SAFA-Gen	*r* = 0.158	*r* = 0.086	*r* = 0.097	*r* = 0.053
	*p* = 0.151	*p* = 0.437	*p* = 0.381	*p* = 0.633
SAFA-A So	*r* = 0.195	*r* = 0.086	*r* = 0.078	*r* = 0.069
	*p* = 0.075	*p* = 0.435	*p* = 0.481	*p* = 0.530
SAFA-A Se	*r* = 0.283	*r* = 0.047	*r* = −0.033	*r* = 0.088
	*p* = 0.009*	*p* = 0.674	*p* = 0.768	*p* = 0.426
SAFA-A Sc	*r* = 0.264	*r* = 0.147	*r* = 0.128	*r* = 0.214
	*p* = 0.015*	*p* = 0.183	*p* = 0.246	*p* = 0.050*

SAFA-S Tot	*r* = 0.131	*r* = 0.083	*r* = 0.061	*r* = 0.073
	*p* = 0.234	*p* = 0.451	*p* = 0.582	*p* = 0.507
SAFA-S Som	*r* = 0.155	*r* = 0.071	*r* = 0.060	*r* = 0.102
	*p* = 0.159	*p* = 0.521	*p* = 0.587	*p* = 0.355
SAFA-S Hyp	*r* = 0.136	*r* = 0.208	*r* = 0.169	*r* = 0.054
	*p* = 0.216	*p* = 0.057	*p* = 0.124	*p* = 0.625

SAFA-D Tot	*r* = 0.099	*r* = 0.014	*r* = 0.073	*r* = 0.082
	*p* = 0.372	*p* = 0.901	*p* = 0.510	*p* = 0.459
SAFA-D Dep	*r* = 0.062	*r* = −0.015	*r* = 0.031	*r* = 0.091
	*p* = 0.573	*p* = 0.889	*p* = 0.779	*p* = 0.409
SAFA-D Anhe	*r* = 0.070	*r* = −0.013	*r* = 0.040	*r* = 0.063
	*p* = 0.527	*p* = 0.905	*p* = 0.719	*p* = 0.572
SAFA-D Irrit	*r* = 0.161	*r* = 0.032	*r* = 0.080	*r* = 0.164
	*p* = 0.143	*p* = 0.771	*p* = 0.468	*p* = 0.136
SAFA-D Inad	*r* = −0.026	*r* = 0.120	*r* = 0.173	*r* = 0.086
	*p* = 0.817	*p* = 0.279	*p* = 0.116	*p* = 0.435
SAFA-D Insec	*r* = 0.056	*r* = 0.122	*r* = 0.129	*r* = −0.039
	*p* = 0.633	*p* = 0.296	*p* = 0.270	*p* = 0.737
SAFA-D Guil	*r* = 0.283	*r* = 0.032	*r* = 0.130	*r* = 0.235
	*p* = 0.014*	*p* = 0.784	*p* = 0.270	*p* = 0.044*
SAFA- Hop	*r* = 0.076	*r* = 0.120	*r* = 0.113	*r* = −0.039
	*p* = 0.517	*p* = 0.307	*p* = 0.336	*p* = 0.739

*p ≤ 0.05.

TAS-F1, “Difficulty identifying feelings,” TAS-F2, “Difficulty describing feelings” and TAS-F3 “Externally oriented thinking”; SAFA, Psychiatric scales for self-administration for youths and adolescents; SAFA-A Ge, “Generalized anxiety”; SAFA-A So, “Social anxiety”; SAFA-A Se, “Separation anxiety”; SAFA-A Sc, “School anxiety”; SAFA-A Tot, “Total anxiety”; SAFA-S So, “Somatic symptoms” subscale; SAFA-S Hy, “Hypochondria”; SAFA-S Tot, “Total Somatization,” SAFA-D De, “Depression”; SAFA-D Anhe, “Anhedonia”; SAFA-D Irrit, “Irritability”; SAFA-D Inad, “Sense of inadequacy”; SAFA-D Insec, “Insecurity”; SAFA-D Guil, “Sense of guilty”; SAFA-D Hop, “Hopelessness.”

**Table 7:** Correlation between ASQ and SAFA-A, S, and D.

	ASQ-F1	ASQ-F2	ASQ-F3	ASQ-F4	ASQ-F5
SAFA-A Tot	*r* = −0.126	*r* = 0.052	*r* = 0.038	*r* = 0.057	*r* = −0.048
	*p* = 0.254	*p* = 0.642	*p* = 0.730	*p* = 0.604	*p* = 0.667
SAFA-Gen	*r* = −0.032	*r* = −0.016	*r* = −0.082	*r* = −0.070	*r* = −0.134
	*p* = 0.773	*p* = 0.884	*p* = 0.461	*p* = 0.526	*p* = 0.223
SAFA-A So	*r* = −0.077	*r* = −0.037	*r* = −0.053	*r* = −0.034	*r* = 0.004
	*p* = 0.487	*p* = 0.741	*p* = 0.966	*p* = 0.762	*p* = 0.974
SAFA-A Se	*r* = −0.094	*r* = 0.071	*r* = −0.005	*r* = 0.116	*r* = 0.051
	*p* = 0.393	*p* = 0.521	*p* = 0.966	*p* = 0.295	*p* = 0.644
SAFA-A Sc	*r* = −0.214	*r* = 0.141	*r* = 0.172	*r* = 0.034	*r* = −0.069
	*p* = 0.050*	*p* = 0.201	*p* = 0.118	*p* = 0.760	*p* = 0.532

SAFA-S Tot	*r* = −0.069	*r* = −0.004	*r* = 0.074	*r* = 0.113	*r* = −0.011
	*p* = 0.533	*p* = 0.971	*p* = 0.504	*p* = 0.307	*p* = 0.921
SAFA-S Som	*r* = −0.059	*r* = 0.002	*r* = 0.064	*r* = 0.097	*r* = −0.012
	*p* = 0.597	*p* = 0.986	*p* = 0.565	*p* = 0.379	*p* = 0.913
SAFA-S Hyp	*r* = −0.143	*r* = −0.023	*r* = 0.044	*r* = −0.017	*r* = −0.033
	*p* = 0.193	*p* = 0.838	*p* = 0.689	*p* = 0.879	*p* = 0.767

SAFA-D Tot	*r* = −0.032	*r* = −0.060	*r* = 0.014	*r* = 0.066	*r* = 0.051
	*p* = 0.770	*p* = 0.588	*p* = 0.897	*p* = 0.549	*p* = 0.642
SAFA-D Dep	*r* = −0.011	*r* = −0.069	*r* = 0.038	*r* = 0.122	*r* = −0.033
	*p* = 0.922	*p* = 0.531	*p* = 0.734	*p* = 0.268	*p* = 0.767
SAFA-D Anhe	*r* = 0.104	*r* = −0.008	*r* = −0.035	*r* = 0.014	*r* = 0.051
	*p* = 0.344	*p* = 0.943	*p* = 0.755	*p* = 0.898	*p* = 0.642
SAFA-D Irrit	*r* = −0.103	*r* = −0.007	*r* = 0.052	*r* = 0.039	*r* = −0.048
	*p* = 0.350	*p* = 0.951	*p* = 0.638	*p* = 0.722	*p* = 0.667
SAFA-D Inad	*r* = −0.103	*r* = −0.007	*r* = 0.009	*r* = 0.196	*r* = 0.050
	*p* = 0.351	*p* = 0.951	*p* = 0.936	*p* = 0.074	*p* = 0.654
SAFA-D Insec	*r* = −0.059	*r* = 0.128	*r* = 0.009	*r* = 0.071	*r* = 0.008
	*p* = 0.618	*p* = 0.246	*p* = 0.936	*p* = 0.547	*p* = 0.945
SAFA-D Guil	*r* = −0.193	*r* = −0.034	*r* = 0.045	*r* = 0.124	*r* = −0.125
	*p* = 0.100	*p* = 0.773	*p* = 0.704	*p* = 0.291	*p* = 0.287
SAFA- Hop	*r* = −0.130	*r* = 0.052	*r* = 0.039	*r* = 0.118	*r* = 0.001
	*p* = 0.271	*p* = 0.660	*p* = 0.742	*p* = 0.316	*p* = 0.990

*p ≤ 0.05.

ASQ-F1, “Confidence in relationships”; ASQ-F2, “Need for approval”; ASQ-F3, “Preoccupation with relationships”; ASQ-F4, “Discomfort with closeness”; ASQ-F5, “Relationships as secondary”; SAFA, Psychiatric scales for self-administration for youths and adolescents; SAFA-A Ge, “Generalized anxiety”; SAFA-A So, “Social anxiety”; SAFA-A Se, “Separation anxiety”; SAFA-A Sc, “School anxiety”; SAFA-A Tot, “Total anxiety”; SAFA-S So, “Somatic symptoms” subscale; SAFA-S Hy, “Hypochondria”; SAFA-S Tot, “Total Somatization,” SAFA-D De, “Depression”; SAFA-D Anhe, “Anhedonia”; SAFA-D Irrit, “Irritability”; SAFA-D Inad, “Sense of inadequacy”; SAFA-D Insec, “Insecurity”; SAFA-D Guil, “Sense of guilty”; SAFA-D Hop, “Hopelessness.”

Our results evidenced an important role of maternal alexithymia levels on their children’s psychological profile, in particular on anxiety symptoms.

## Discussion

This is the first study which examines the role of maternal alexithymia and attachment style on their children’s migraine severity and psychological profile. The main results of our study are as follows: (1) there is no relationship between maternal alexithymia levels, attachment style, and children’s migraine features (severity and frequency); (2) maternal alexithymia shows a relationship with children’s insecure attachment style, while attachment does not; (3) there are correlations between maternal alexithymia and patients’ anxiety symptoms.

### Role of Maternal Alexithymia and Attachment Style on Their Children Migraine Severity and Attachment Style

Childhood migraine is a neurological complex and multifactorial disorder, in which several factors may negatively affect the severity of headache. The present study showed that insecure attachment (88.1%) and, in particular, ambivalent style (42.9%) is very common among children/adolescents suffering from migraine. These data are in accordance with a previous study from our group in which the role of attachment on children’s migraine features and psychological profile were explored (14); in this previous paper, the hypothesis that a dysfunctional relationship between children and their mothers could be a vulnerability factor in young migraineurs was suggested.

While a growing body of literature analyzed the influence of maternal psychological symptoms on children’s headache (39, 46), few studies explored the importance of maternal alexithymia in this disorder (33, 34). To the best of our knowledge, no study focused on maternal attachment style in migrainous children. In a previous paper, Cerutti et al. found higher rates of alexithymia in mothers of adolescents with migraine (33); this study, however, did not consider the effect of maternal alexithymia on patients’ migraine severity.

In the present study, neither maternal alexithymia levels nor the attachment style show a relationship with patients’ migraine severity (intensity and frequency). On the other hand, we found that alexithymia traits show a correlation with children/adolescents’ attachment style. Our results suggest that even if maternal emotional awareness and interpersonal relations have no causative role on children’s migraine severity, it may influence children’s attachment style and their affective regulation. We hypothesize that, in our sample, alexithymic traits may affect mothers’ ability to decode their children’s needs and emotions and to adequately cope with them.

Alexithymia is a personality trait characterized by an impaired ability to identify and communicate emotions of self and others, which may result in ineffective and unempathic emotional responding (47). Mothers with difficulty in expressing feelings and regulating emotions can inhibit their children’s attitude to self-regulate their feelings and emotional state, influencing their body experience, leading to a tendency to somatization (24). On the other hand, patients with ambivalent attachment style may exaggerate the nature of affective signals in order to force their “insensitive” parents to respond to their signals of distress (5, 8). As we discussed in our previous study, these hyperactivation strategies may involve increased expression of distress and catastrophic thinking which can impact on frequency and intensity of childhood migraine (14). Thus, we could speculate that children/adolescents’ attachment style has a mediating role between maternal alexithymia traits and migraine features.

It is difficult to explain the reasons why in our sample only maternal alexithymia levels, but not maternal attachment style, show a relationship with their children’s attachment style. We can hypothesize that this result may be influenced by the nature of the ASQ questionnaire (44, 45). The ASQ is a self-report questionnaire and may be unable to elicit stress and danger situations, necessary to activate the attachment system. Moreover, this tool may only reveal the conscious feelings and perceptions of relationships (48).

### Correlation between Maternal Alexithymia, Attachment Style, and Psychological Symptoms in Children with Migraine

Several studies described how dysfunctional parenting may lead to various psychopathological conditions. Caregivers’ emotional unavailability and incongruous affective responses may undermine the development of children’s patterns of emotional regulation (5, 6, 15).

Data from the literature provided a correlation between unsupportive parental responsiveness and adolescents’ externalizing and internalizing symptoms (49, 50). Mechanisms underlying unsupportive or disconfirming parental responses may be predicted by their difficulties in emotions regulation and alexithymic traits (15, 16, 23, 24).

This is the first study to explore the correlation between maternal attachment style, alexithymia, and children/adolescents’ psychological symptoms (anxiety, depression, and somatization) in a group of patients suffering from migraine.

In our patients, maternal alexithymia levels were associated with separation anxiety, anxiety related to school and children’s feeling of guilt. In particular, our data evidenced a relationship between “Externally-oriented thinking” (TAS-F3), children’s anxiety related to school and feeling of guilt. Moreover, our data showed a negative correlation between maternal confidence in self and in others (ASQ-F1) and patients’ anxiety related to school.

We can hypothesize that mothers may be more focused on superficial aspects and on external events, such as school performance, rather than children’s psychological experiences, affective thinking, and intimate emotional states. Showing difficulties in appreciating other people’s emotions and being empathic, mothers with high levels of alexithymia might appear less responsive to their children’s psychological needs, unconcerned and affectively less involved in the relationship with them. The persistence of these maternal dysfunctional attitudes may increase children’s uncertainty about parental affective involvement and presence, inducing symptoms of separation anxiety and feelings of loss (51). On the other hand, we can suppose that the inclination of alexithymic mothers to enact an externally oriented thinking may lead migraine children and adolescents to performance/school anxiety symptoms, feeling of guilt and a fear of being “unacceptable.” Anxiety related to school can interfere with learning and may lead to poor school performance (52). It can be hypothesized that, in our sample, this may result in low maternal confidence in self and others which in turn can contributes to the maintenance of children’s anxiety levels.

### Limitations

The present study has some limitations that must be taken into account in the interpretation of results. (1) Our data are derived from children/adolescents (and mothers) referred to our tertiary Headache Center and may not be representative of the whole pediatric population suffering from migraine without aura; (2) The psychological tools employed in our study (TAS-20, ASQ, SAFA-A, D, and S) have a self-report nature; although, they have been considered valid for psychological screening, they are not suitable for a formal diagnosis of psychiatric disorder; moreover, ASQ, as a self-report questionnaire, may not be able to elicit stress and danger situations, which are indispensable to activate the attachment system; (3) In future studies, it would be important to further explore not only the role of maternal attachment and alexithymia but also the role of maternal migraine features on their children’s migraine severity, attachment style, and psychological profile.

## Conclusion

This is the first study exploring the role of maternal emotional regulation and relationship bond on their children headache severity in a selected population of children/adolescents with migraine. Our data showed that maternal alexithymia and attachment style have no relationship with children’s migraine severity. However, we found that maternal alexithymia is associated with patients’ attachment style and psychological symptoms (anxiety and feeling of guilt). These results can have therapeutic consequences. Given the high risk among young migraineurs of developing an insecure attachment style and anxiety symptoms, which are known to impact on children/adolescents migraine severity (14), special attention should be paid to maternal alexithymic traits and mother–child interaction.

## Ethics Statement

All participants and their parents gave signed, informed consent to participate to the study. The study was approved by the local ethics committee and was conducted in compliance with the Helsinki Declaration.

## Author Contributions

ST, SG, and MV conceived and supervised the project. ST was responsible for data collection. LP and FB were involved in data analysis. FB, CR, SG, and MP were involved in interpretation and assisted in preparation of the manuscript. ST and MV drafted the manuscript and were the main authors. AR, MD, VV, BB, and FV critically reviewed and revised this manuscript for important intellectual inputs. All authors read and approved the manuscript.

## Conflict of Interest Statement

The authors declare that the research was conducted in the absence of any commercial or financial relationships that could be construed as a potential conflict of interest.

## Abbreviations

IWM, internal working models; CHD-III-beta, Headache Classification Committee of the International Headache Society; HF, high frequency; LF, low frequency; MP, mild pain; SP, severe pain; SAT, separation anxiety test; SAFA, psychiatric scales for self-administration for youths and adolescents; SAFA-A, SAFA-anxiety; SAFA-D, SAFA-depression; SAFA-S, SAFA-somatization; TAS-F1, difficulty identifying feelings; TAS-F2, difficulty describing feelings; TAS-F3, externally oriented thinking; ASQ-F1, confidence in relationships; ASQ-F2, need for approval; ASQ-F3, preoccupation with relationships; ASQ-F4, discomfort with closeness; ASQ-F5, relationships as secondary.
